# *Clonorchis sinensis* infection induces hepatobiliary injury *via* disturbing sphingolipid metabolism and activating sphingosine 1-phosphate receptor 2

**DOI:** 10.3389/fcimb.2022.1011378

**Published:** 2022-10-19

**Authors:** Ji-Xin Liu, Man Liu, Guo-Zhi Yu, Qian-Qian Zhao, Jian-Ling Wang, Yan-Hong Sun, Stephane Koda, Beibei Zhang, Qian Yu, Chao Yan, Ren-Xian Tang, Zhi-Hua Jiang, Kui-Yang Zheng

**Affiliations:** ^1^ Jiangsu Key Laboratory of Immunity and Metabolism, Laboratory of Infection and Immunity, Department of Pathogenic Biology and Immunology, Xuzhou Medical University, Xuzhou, China; ^2^ Department of Clinical Pathogen Biology, Qiqihaer Medical University, Qiqihaer, China; ^3^ Department of Pathogen Biology, Qiqihaer Medical University, Qiqihaer, China; ^4^ Institute of Parasitic Disease Control and Prevention, Guangxi Key Laboratory for the Prevention and Control of Viral Hepatitis, Guangxi Zhuang Autonomous Region Center for Disease Control and Prevention, Nanning, China

**Keywords:** *Clonorchis sinensis*, hepatobiliary injuries, biliary epithelial cells (BECs), sphingolipid metabolism, sphingosine 1-phosphate receptor 2

## Abstract

*Clonorchis sinensis (C. sinensis)* infection induces severe hepatobiliary injuries, which can cause inflammation, periductal fibrosis, and even cholangiocarcinoma. Sphingolipid metabolic pathways responsible for the generation of sphingosine-1-phosphate (S1P) and its receptor S1P receptors (S1PRs) have been implicated in many liver-related diseases. However, the role of S1PRs in *C. sinensis*-mediated biliary epithelial cells (BECs) proliferation and hepatobiliary injury has not been elucidated. In the present study, we found that *C. sinensis* infection resulted in alteration of bioactive lipids and sphingolipid metabolic pathways in mice liver. Furthermore, S1PR2 was predominantly activated among these S1PRs in BECs both *in vivo* and *in vitro*. Using JTE-013, a specific antagonist of S1PR2, we found that the hepatobiliary pathological injuries, inflammation, bile duct hyperplasia, and periductal fibrosis can be significantly inhibited in *C. sinensis*-infected mice. In addition, both *C. sinensis* excretory-secretory products (CsESPs)- and S1P-induced activation of AKT and ERK1/2 were inhibited by JTE-013 in BECs. Therefore, the sphingolipid metabolism pathway and S1PR2 play an important role, and may serve as potential therapeutic targets in hepatobiliary injury caused by *C. sinensis*-infection.

## Introduction

*Clonorchis sinensis (C. sinensis)* is a key food-borne parasite that is widely prevalent in China, Korea, Vietnam, and the far eastern part of Russia, infecting about 15 million people (Qian et al., 2016). The worm mainly lives in the bile duct and causes numerous pathophysiological changes, such as inflammation (Kim et al., 2017), hyperplasia of epithelial cells (Won et al., 2019), metaplasia in the mucosa (Choi et al., 2015), and especially severe biliary injuries, which leads to cholangitis, periductal fibrosis, cirrhosis in advanced cases, and even cholangiocarcinoma (Uddin et al., 2015; Lu et al., 2018; Yan et al., 2021). It has been reported that the carcinogenic liver fluke causes ~5000 human cholangiocarcinoma cases annually (Qian and Zhou, 2021). The pathogenesis of *C. sinensis* infection encompasses several factors, such as mechanical obstruction of worms in the bile duct, mechanical injury of the biliary mucosal barrier system, and immunopathology caused by infection-related inflammation which includes secondary bacterial infection, direct chemical irritation, toxicity, and immune damage of excretion/secretion of products (CsESPs) (Qian et al., 2016). However, the pathogenesis of clonorchiasis remains obscure.

Biliary epithelial cells (BECs), also called cholangiocytes, lining the extra- and intrahepatic bile ducts, are highly specialized cells. It not only participates in bile production and homeostasis, but also can be activated and involved in inflammation by secreting chemokines and cytokines. It also can directly regulate the biology of myofibroblasts, which are responsible for collagen deposition within the liver (Banales et al., 2019). *C. sinensis* dwells in the bile duct, and both the worm itself and CsESPs are in full contact with BECs. Therefore, focusing on BECs is of far-reaching significance to further explore the pathogenic mechanism of *C. sinensis* infection. Upon injury, activated BECs expand, forming a transient luminal epithelium and establishing an auxiliary biliary system in a process known as a ductular reaction (DR) (Planas-Paz et al., 2019), which is the most common in *C. sinensis* infection. DR is characterized by the proliferation of reactive bile ducts induced by various liver injuries (Sato et al., 2019). In addition, it may be induced by cholestasis (Meadows et al., 2021) or parasites and their ESPs within the bile duct (Gieseck et al., 2016; Yan et al., 2021). It can alter the microenvironment around the duct by secreting various cytokines, chemokines, and growth factors, triggering and perpetuating inflammatory and profibrotic responses (Guicciardi et al., 2020). However, the mechanisms underlying the interaction between *C. sinensis* (including CsESPs) and BECs caused chronic hepatobiliary injuries are largely unknown.

Sphingolipids, a bioactive metabolite, not only serve as the lipid components of biological membranes but also act as crucial molecules involved in regulating cell growth, survival, immune cell transport, as well as vascular and epithelial integrity, which are particularly important in inflammation and cancer, and play a key role in multiple stages of many diseases (Maceyka and Spiegel, 2014). Both around 40 biosynthesis enzymes and the corresponding metabolites in their pathway can affect bioactive sphingolipids in mammals (Hannun and Obeid, 2018). Sphingolipids reach the lysosomal compartment, where they are sequentially degraded into ceramide, which is the core component of all complex sphingolipids including sphingomyelin and glycosphingolipids, which are then degraded to sphingine by ceramides. Sphingosine can be regenerated into ceramides by the recycling pathway or phosphorylated by sphingosine kinases (Sphk1 and Sphk2) to form S1P (Green et al., 2021). Sphingosine 1-phosphate (S1P), a product of membrane sphingolipid metabolism, is secreted and acts through G protein-coupled S1P receptors (has five subtypes, including S1PR1, S1PR2, S1PR3, S1PR4, and S1PR5) in vertebrates. S1PR signaling regulates multiorgan pathophysiological processes (Cartier and Hla, 2019). However, the expression of S1PRs is significantly different in different types of tissues (Nagahashi et al., 2016).

S1PR2, also known as EDG-5, is ubiquitously expressed in [dendritic cells (Park and Im, 2020), macrophages (Hou et al., 2020), lymphocytes (Xiong et al., 2019)], smooth muscle cells (Liu et al., 2020a), cardiomyocytes (Chen et al., 2018a), hepatocytes (Webster and Anwer, 2016), and intestinal epithelial cells (Chen et al., 2018b). S1PR2 has diverse functions and has been implicated in many organ-system pathologies, for example, regulating the blood-brain barrier and intestinal barrier permeability (Chen et al., 2018b; Wan et al., 2018), regulating hepatic lipid metabolism (Kwong et al., 2015), suppressor in colorectal cancer (Petti et al., 2020), activating different signaling pathways and thus exerting different biological effects such as MAPK signaling pathway (Hou et al., 2021a), ERK1/2, JNK signaling pathway (Sapkota et al., 2019), and PI3K/AKT signaling pathway (Chen et al., 2017). JTE-013, a potent and selective S1PR2 antagonist, could exert the inhibitory effect of S1PR2 on a variety of cells, which could also play a different role by mediating multiple signaling pathways (Wang et al., 2017). A recent study has shown that S1PR2 expression is elevated in BECs, and S1PR2 is activated by conjugated bile acid, inducing BECs proliferation (Wang et al., 2017), promoting cholangiocarcinoma cell invasive growth (Liu et al., 2014). However, whether S1PR2 plays a role in the hepatobiliary duct injury caused by *C. sinensis* infection or not remains unclear.

In the present study, we found that the activation of some sphingolipid metabolism-related kinases, especially Sphk, could promote the generation of S1P which may act as endogenous ligand of S1PR2 during *C. sinensis* infection *in vivo*. Inhibition of S1PR2 could alleviate hepatobiliary damage, decrease pro-inflammatory cytokines expression, abate periductal fibrosis, and reduce biliary injuries *in vivo*. Moreover, the expression of S1PR2 in BECs was significantly increased after *C. sinensis* infection. At the same time, our study showed that the expression of S1PR2 was significantly increased in the CsESPs-treated BECs, and the inhibition of S1PR2 decreased the expression of pro-inflammatory cytokines and AKT/ERK signaling pathway. This study suggests that the activation of S1PR2 promotes hepatobiliary duct injury induced by *C. sinensis* infection. Therefore, our present study may provide clues for the finding of new strategies for the prevention and therapy of clonorchiasis.

## Material and methods

### Ethics statement

According to route the Guidelines for Animal Experiments of Xuzhou Medical University and the National Guide for the Care and Use of Laboratory Animals, the authors are accountable for all animals’ experimental procedures and the study protocol was reviewed and approved under a project license (No. 201701w007) by the Committee on Ethics of Animal Experiments, Xuzhou Medical University.

### Preparation of *C. sinensis* metacercariae

The preparation refers to the previously described (Yu et al., 2016; Dai et al., 2020). Briefly, *Pseudorasbora parva* (a kind of freshwater fish) obtain from endemic areas of *C. sinensis* infection, and screen out the positive fish after morphological identification. The meat was stripped and chopped, and then digested with artificial gastric juice, a solution of 0.7% pepsin A (1:3000) and 0.1% HCl, at 37°C in a shaking water bath for 12h. The digested mixture was filtered through a 100mm sieve. Then the pellet was sedimented in Alsever’s juice (a metacercariae preservation solution) in a sedimentation jar until the supernatant was clear. *C. sinensis* metacercariae were identified by morphological characteristics, collected under a dissecting microscope, and then stored at 4°C until use.

### Preparation of *C. sinensis* excretory-secretory products

CsESPs were prepared as described elsewhere (Kim et al., 2021; Yan et al., 2021). In brief, 8-week-old white guinea pigs were individually infected with 200 C*. sinensis* metacercariae. Conventional feeding for 8 weeks, and then euthanized, the *C. sinensis* -infected livers were extracted. The adult worms of *C. sinensis* were collected as possible from the bile ducts. The following operations need to be completed in the ultra-clean table. Collected worms were washed five times with sterile 1×phosphate-buffered saline (1×PBS) containing 1% (v/v) *penicillin/streptomycin* (*P/S*, Hyclone, USA), the cultured proportion is 100 worms/10ml medium in a dish, then gently shake the dish to evenly distribute the worms and followed by incubation for 24h at 37°C with 5% CO_2_ incubator. The medium was collected and centrifuged for 30min at 1500×g to remove eggs. After that, the medium was centrifuged a second time for 30min at 3500×g to remove cellular debris, and for the third time for 30min at 12000×g to remove spermine, and then the culture supernatant was filtered with a syringe-driven 0.22μm filter, CsESPs was obtained. The concentration of CsESPs was measured using Enhanced BCA Protein Assay Kit (Beyotime, Shang-hai, China) and stored at -80°C for further use.

### Animal experiments

#### *C. sinensis* -infected animal model construction and JTE-013 administration

All mice were bred in pathogen-free conditions with normal lighting, and both male and female mice were used. Following a simple randomization procedure, 6~8 weeks old BALB/C mice were randomly assigned to the control group (n=8) and *C. sinensis*-infected groups (n=8), and randomly selected mice (n=8) from each group for JTE-013 treatment. Infection was carried out at a dose of 50 metacercariae per mouse by gavage in *C. sinensis*-infected groups. The control group was treated with PBS in the same way. JTE-013 (Cayman Chemical (Boston, MA), Cat No.10009458) was administered intraperitoneally at 10mg/kg twice a week for 4 weeks.

#### Hepatobiliary function serological test

The activities of alanine aminotransferase (ALT), aspartate aminotransferase (AST), alkaline phosphatase (ALP), total bilirubin (TBIL), direct bilirubin (DBIL), total bile acid (TBA), cholesterol (CHO) and triglyceride (TG) in the serum were detected by a fully automatic biochemical analyzer to indicate the hepatobiliary function in mice in the Laboratory Department of the Affiliated Hospital of Xuzhou Medical University.

#### Histology, immunohistochemistry, and immunofluorescence staining

To evaluate the histopathological changes in *C. sinensis*-infected mice and their capacity to induce bile duct damage, the hepatobiliary tissue was serially sectioned at 4µm for H&E and Alcian Blue staining according to the manufacturer’s instructions (Wuhan service Biotechnology Research Institute, China). After sealing the slides with neutral adhesive, the pathological changes of the stained histological sections were observed by microscope (Olympus, Japan). The hyperplasia, mucin secretion, damage to the BECs, infiltration of inflammatory cells, and histological scoring were evaluated based on the histological findings. For IHC and IF staining, serial sections of embedded tissue from each mouse were used for the staining of S1PR2 and cytokeratin 19 (CK19). The hepatobiliary tissue was deparaffinized, hydrated, and heated in citric acid buffer at 95°C for 15min and then blocked with 5% BSA for 30min. The slides were then incubated overnight with primary Anti-S1PR2 (1:100, sc-365963 Santa Cruz Biotechnology, Inc., Santa Cruz, CA), Anti-Cytokeratin 19 (1:200, ab52625, Abcam, Cambridge, USA). After washing with PBS, DAB (1:200, ZSGB–BIO, Beijing, China) as an enzyme-substrate was added. Five high-power fields (×200 magnifications, Olympus, Japan) were randomly selected from the stained section. CK19 and S1PR2-positive expressions integrated optical density IOD (Integral optical density) was calculated by Image-Pro Plus 6.0 software. The higher the IOD value, the stronger the positive expression.

#### Sirius red staining

The hepatobiliary duct tissues were routinely fixed, embedded, and sectioned using the same method as HE staining, and then stained with Sirius Red, according to the manufacturer’s instructions (Servicebio, Wuhan, Hubei, China). The sections were observed under a microscope and digitized using an imaging system (Olympus, Japan). The selection of the high magnification field of view and the calculation method of the integral optical density (IOD) of fibrous tissue is the same as above.

#### Measurement of hepatic hydroxyproline content

To quantify hepatobiliary fibrosis, hepatic hydroxyproline content was determined according to the instructions of the manufacturer (Nanjing jiancheng Institute of Biotechnology, Nanjing, China) according to the manufacturer’s recommendations. HYP content was measured at 550nm using an HYP standard curve. The value of the hepatic HYP level was expressed as μg/g wet tissue.

#### RNA isolation and quantitative real-time PCR

Total RNA was extracted from hepatobiliary tissue and BECs by using TRIzol reagent according to the manufacturer’s instructions (Invitrogen, Carlsbad, CA, USA), and then reverse transcribed to complementary DNA. Quantitative real-time PCR was performed using SYBR green real-time PCR master mix according to the manufacturer’s instructions (PrimeScript™ RT reagent Kit and TB Green^®^ Premix Ex Taq™, Takara, Japanese). Relative quantification of each gene expression was calculated in terms of the comparative cycle threshold (Ct) normalized by β-actin (Nanjing General Biotech Co. China). Expression levels of selected genes (for primers, see **Supplementary Table S1**).

#### Enzyme-linked immunosorbent assay

Cell culture supernatant and hepatobiliary tissue homogenate from each mouse were immediately subjected to evaluate the concentrations of IL-1β, TNF-α, and IL-6 by a commercial ELISA Kit with Plates (Thermo Scientific, USA). All procedures were performed according to the instructions provided by the kit. Concentrations of cytokines in the cell culture supernatant and hepatobiliary tissue homogenate were calculated using standard curves as references. In addition, hepatobiliary tissue homogenate from each mouse was immediately subjected to evaluate the concentration of S1P by a commercial ELISA Kit with Plates (Lanpaibio, LP-M05715, Shanghai, China). The procedures were performed according to the instructions provided by the kit. Concentrations of S1P in the hepatobiliary tissue homogenate were calculated using standard curves as references.

#### Western blotting

Total protein was extracted from BECs and hepatobiliary tissue by using a homogenizer and analyzed with a bicinchoninic acid (BCA) protein concentration assay kit (Beyotime, Shang-hai, China). Anti-phospho-AKT (P-AKT, 1:1000, ab192623, abcam, Cummings Park, USA), Anti-AKT (AKT, 1:2000, ab32505, abcam, Cummings Park, USA), Anti-phospho-ERK1/2 (P-ERK1/2, 1:1000, ab201015, abcam, Cummings Park, USA), Anti-ERK1/2 (ERK1/2, 1:10000, ab184699, abcam, Cummings Park, USA), Anti-EDG1 (EDG1, 1:1000, ab233386, abcam, Cummings Park, USA), Anti-S1PR2 (1:1000, ab235919, abcam, Cummings Park, USA), Anti-EDG3 (EDG3, 1:2000, ab108370, abcam, Cummings Park, USA), Anti-CK19 (CK19, 1:50000, ab52625, abcam, Cummings Park, USA), Anti-α-SMA (α-SMA, 1:2000, ab32575, abcam, Cummings Park, USA), Anti-Sphk1 (Sphk1, 1:500, 10670-1-AP, Proteintech, Wuhan, Hubei, China), Anti-Sphk2 (Sphk2, 1:1000, 17096-1-AP, Proteintech, Wuhan, Hubei, China), and Anti-GAPDH (1:1000, AC033, ABclonal, Cummings Park, USA) were used as primary antibody after separation by electrophoresis in 7.5 and 10% SDS-PAGE with a Bio-Rad electrophoresis system (Hercules, CA, USA). The secondary antibodies (Anti-rabbit IgG, 1:5000, HRP-S0001, Affinity Biosciences, Cincinnati, USA, and Anti-mouse IgG, 1:5000, 7076S, CA, USA) were incubated for 2h at room temperature. The membrane containing antibody-protein complexes was visualized with an enhanced chemiluminescence detection system on radiographs film (Bio-rad, Hercules, CA, USA). The bands were scanned and analyzed by the software Quantity ONE (Bio-rad, Hercules, CA, USA). The expression of the protein in each sample was normalized by GAPDH or beta-actin (Santa Cruz Biotechnology, CA, USA).

#### Metabolomics analysis

Metabolic profiling performed in the present study included sample preparation, metabolite extraction, and LC/MS analysis. All liver samples and QC samples were used for metabolomic analysis by the UHPLC system. Detailed method descriptions are provided in Supplementary Information (**Supplementary Materials and Methods**).

#### Cell culture

A well-characterized human BECs, HIBEpiC (human intrahepatic biliary epithelial cells), was commercially available from Shanghai Zhong Qiao Xin Zhou Biotechnology Co., Ltd (ZQ0890, Biotechnology, Shanghai, China). The cells were cultured and maintained in Epithelial Cell Complete Medium, which contains Basal medium (ZQ-1315): Fetal Bovine Serum (FBS, Cat. No. 0010): epithelial growth factor (EpiCGS, Cat. No. 4152): *Penicillin&Streptomycin* solution (*P/S*, Cat. No. 0503) = (100:2:1:1), at 37°C with 5% CO_2_ in a humidified cell-culture incubator. For the S1P, CsESPs, and JTE-013 stimulation experiment, the cells were firstly starved for 6h in a Basal medium without FBS and EpiCGS, but containing *P/S*, and then the cells maintained in Epithelial Cell Complete Medium, were stimulated using S1P (100nM), CsESPs (60μg/ml) and JTE-013 (10μM). The cells were pretreated with JTE-013 for 30min, then treated with medium, S1P or CsESPs, and incubated at 37°C for 24h. The cells and culture supernatant were harvested for further use.

#### Cell invasion assays

BECs were seeded in the Matrigel-coated upper chamber. Cells were pretreated with JTE-013 (10μM) for 30min, then treated with medium, S1P (100nM) or CsESPs (60μg/ml) incubated at 37°C for 12h. Stained with 0.1% crystal violet solution cells were counted and analyzed as described previously (Liu et al., 2014).

### Statistical analysis

All the data were expressed as mean ± SEM. The data were analyzed by SPSS 19.0 software (SPSS Inc, Chicago, IL, USA). The student *t*-test was used for comparison between the two groups. One-way ANOVA with Tukey’s *post hoc* test was used for multiple comparisons. *P*<0.05 was considered statistically significant.

## Results

### Lipid metabolism changes after *C. sinensis* infection by metabolomics analysis

**Figure 1:**
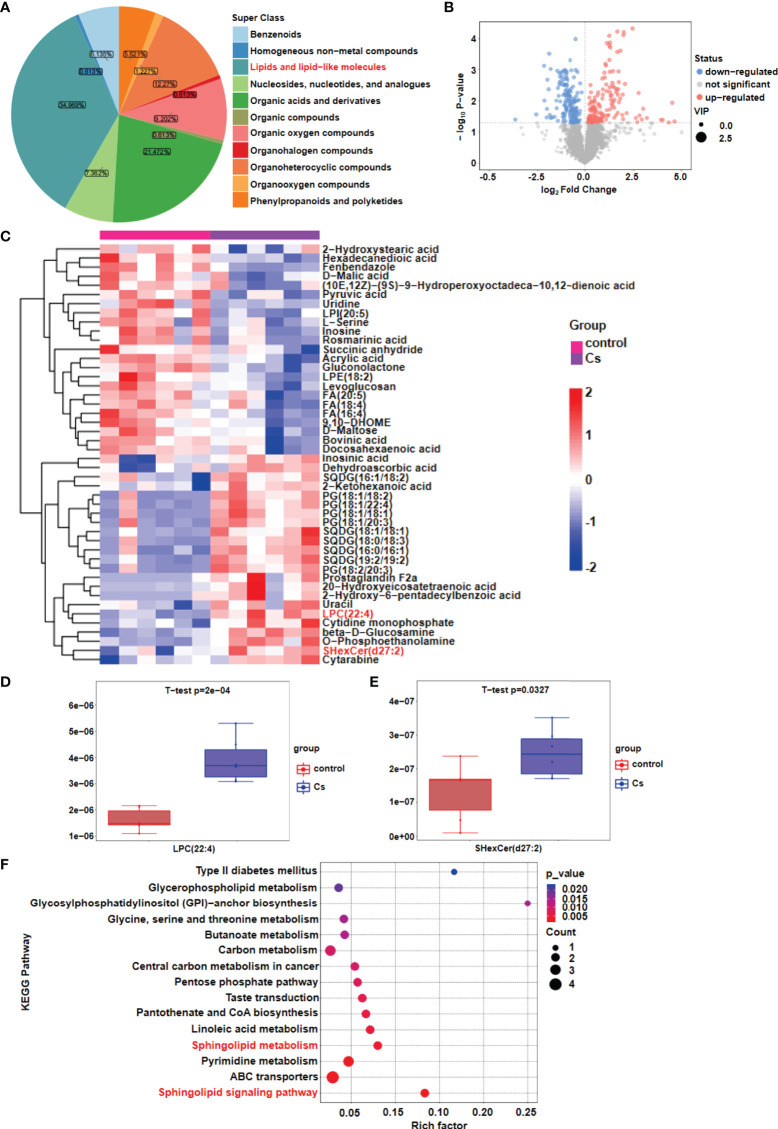
Lipid metabolism changes after *C. sinensis* infection. **(A)** Pie plot of metabolite classification and proportion. **(B)** Volcano plot for the *C. sinensis*-infected group vs the control group by metabolomics. **(C)** Heatmap of hierarchical clustering analysis for the *C. sinensis*-infected group vs the control group by metabolomics. **(D)** Boxplot displays a quantitative analysis of metabolite content of LPC for the *C. sinensis*-infected group vs the control group by metabolomics. **(E)** Boxplot displays a quantitative analysis of metabolite content of SHexCer for the *C sinensis*-infected group vs the control group by metabolomics. **(F)** KEGG Pathway Analysis for the *C. sinensis*-infected group vs the control group by metabolomics.

To investigate lipid dyshomeostasis during the *C. sinensis* infection in hepatobiliary tissue of BALB/C mice, differential metabolites were analyzed by metabolomics. Our results showed that lipid metabolism is the most significant metabolic pathway in the liver after *C. sinensis* infection (**Figure 1A**). The global distribution of the differential metabolites is displayed by a volcano plot, 147 metabolites were upregulated and 165 metabolites were downregulated in the liver of *C. sinensis*-infected mice, compared with those in the non-infected mice (**Figure 1B**). Heatmaps illustrate the 46 metabolites with the highest differences in relative abundance between *C. sinensis*-infected groups and control groups (**Figure 1C**). We found that Lysophosphatidylcholine (LPC), a pro-inflammatory lipid, was significantly higher, and further experiments revealed that the *Lpcat4* and *Pla2g4f*, enzymes that regulate LPC biosynthesis, significantly increased the gene expression levels (**Figure 1D**, *P*<0.001; **Supplementary Figure 1A**, *P*<0.001). Additionally, SHexCer, a sphingolipid metabolite, was significantly higher in the *C. sinensis-*infected group than that in non-infected mice (**Figure 1E**, *P*<0.05). Furthermore, metabolomics pathway enrichment analysis showed that these different expressed genes (DEGs) were mainly enriched in the sphingolipid metabolic and sphingolipid signaling pathway (**Figure 1F**). Triglycerides and cholesterol as lipid components were also tested, and the results showed no change in triglycerides (TG) after *C. sinensis* infection (**Supplementary Figure 1C**) but the elevated cholesterol levels was found in *C. sinensis*-infected mice, compared with non-infected mice (**Supplementary Figure 1D**, *P*<0.05).

### *C. sinensis* infection activates sphingolipid metabolic pathways for the generation of S1P in hepatobiliary tissue of BALB/C mice

**Figure 2:**
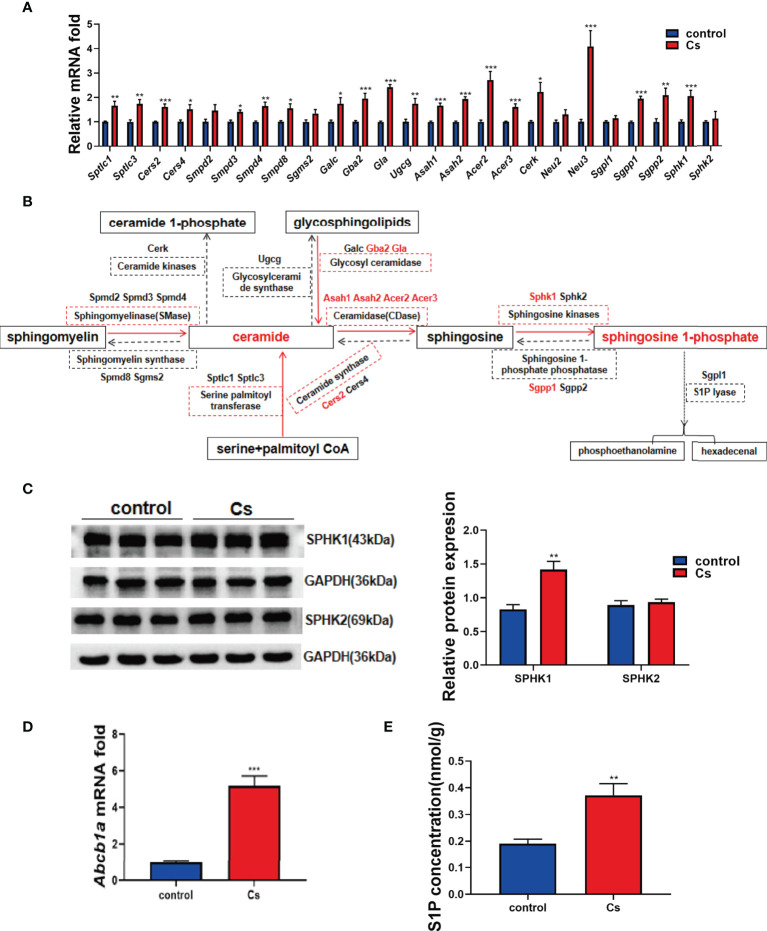
*C. sinensis* infection resulted in activation of sphingolipid metabolic pathways. **(A)** qRT-PCR assays were performed to measure the mRNA levels of enzymes involved in sphingolipid metabolic pathway, including *Sptlc1, Sptlc3, Cers2, Cers4, Smpd2, Smpd3, Smpd4, Samd8, Sgms2*, *Galc, Gba2, Gla*, *Ugcg, Asah1, Asah2, Acer2, Acer3, Cerk, Neu2, Neu3, Sgpl1, Sgpp1, Sgpp2, Sphk1, and Sphk2*. **(B)** The results schemed as a diagram. **(C)** Western blotting analysis of the protein expression levers of Sphk1 and Sphk2 in the liver of control and *C. sinensis*-infected mice. **(D)** The gene expression levels of ABC transporter protein. Measurement of the *Abcb1a* relative mRNA expression in liver tissue of control and *C. sinensis* infected mice. **(E)** The concentration of S1P in liver was determined by ELISA in control and *C. sinensis*-infected mice. Compared with control group, *P<0.05, **P<0.01, ***P<0.001.

Given that sphingolipids were altered more significant than other lipid classes in the *C. sinensis*-infected hepatobiliary tissue, we further defined the possible mechanism by which *C. sinensis* infection altered the levels of sphingolipids. By qRT-PCR, we found that *C. sinensis* infection markedly upregulated the expression of metabolizing enzymes of sphingolipids, such as *Cers2, Gba2*, *Gla*, *Asah1*, *Asah2*, *Acer2*, *Acer3*, *Neu3*, *Sgpp1*, and *Sphk1* (**Figure 2A**, *P*<0.001). The results were schemed as a diagram (**Figure 2B**). It is worth noting that *Sphk1* was one of upregulated enzymes in terms of the fold change in mRNA levels. Moreover, we confirmed that *C. sinensis* infection indeed increased Sphk1 protein levels in hepatobiliary tissue of *C. sinensis*-infected mice, compared with non-infected mice (**Figure 2C**, *P*<0.01). Additionally, the mRNA levels of *Sphk2*, an isozyme of Sphk1 and protein levels were slightly regulated but no statistical change in *C. sinensis*-infected hepatobiliary tissue, compared with non-infected mice (**Figure 2C**, *P*>0.05). Interestingly, the mRNA levels of *Abcb1a*, an ABC transporter that can transfer S1P from intracellular to extracellular, were also significantly upregulated in *C. sinensis*-infected hepatobiliary tissue than that in non-infected mice (**Figure 2D**, *P*<0.001). Notably, the intrahepatic S1P concentration was markedly increased in *C. sinensis*-infected hepatobiliary tissue, compared with that in the non-infected mice (**Figure 2E**, *P*<0.01).

**Figure 3:**
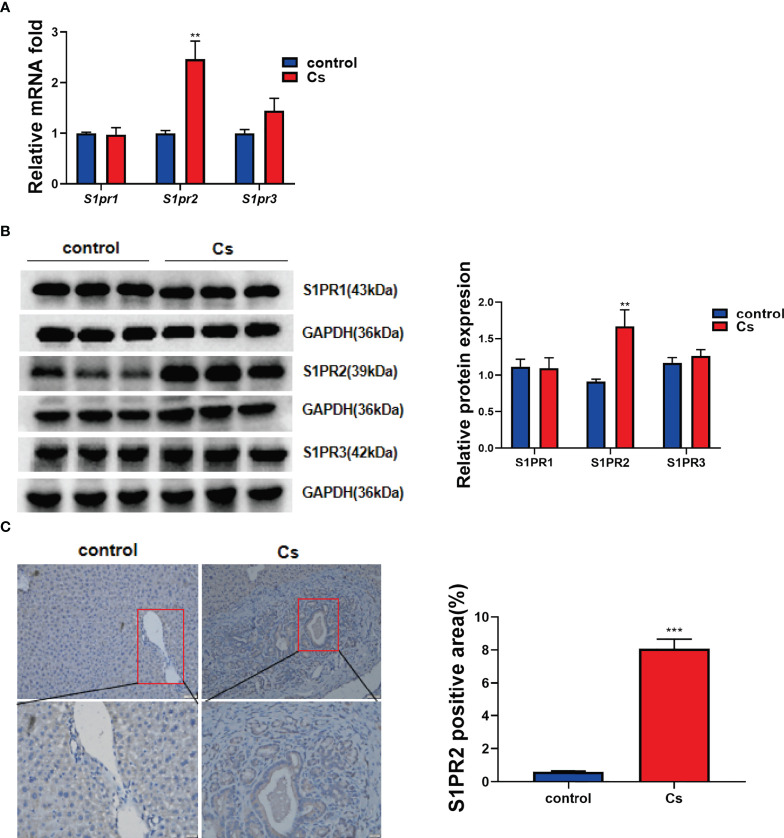
*C. sinensis* infection significantly increased S1PR2 expression. **(A)** The relative mRNA expression for *S1pr1*, *S1pr2 and S1pr3* in hepatobiliary tissue of control and *C. sinensis*-infected mice. **(B)** The relative protein expression for S1PR1, S1PR2 and S1PR3 in liver of control and *C. sinensis*-infected mice. **(C)** The expression of S1PR2 in liver of control and *C. sinensis*-infected mice by immunofluorescence assay. Compared with control group, **P<0.01, ***P<0.001.

Previous studies reported that S1PR2 was the predominant S1PR in hepatocytes and cholangiocarcinoma (CCA) cells (HuCCT1, CCLP1 cell line), and could be activated by S1P (Studer et al., 2012; Liu et al., 2014). We determined whether S1PR2 is involved in *C. sinensis* infection-induced hepatobiliary injury or not. Firstly, the expression levels of different S1PRs in hepatobiliary tissue were examined. As results shown in **Figure 3A**, *S1pr2* increased significantly in infected mice. However, *S1pr1* and *S1pr3* remained unchanged (**Figure 3B**, *P<*0.01). Consistent with real-time RT-PCR data, the western blotting analysis indicated that the levels of S1PR2 but not S1PR1 and S1PR3 were dramatically elevated in *C. sinensis*-infected liver, compared with non-infected mice (**Figure 3B**, *P*<0.01). Additionally, the S1PR2 expressions were indeed increased as measured by IHC in the biliary tissue (**Figure 3C**, *P*<0.001).

### Inhibition of S1PR2 alleviates hepatobiliary damage in mice infected with *C. sinensis*

**Figure 4:**
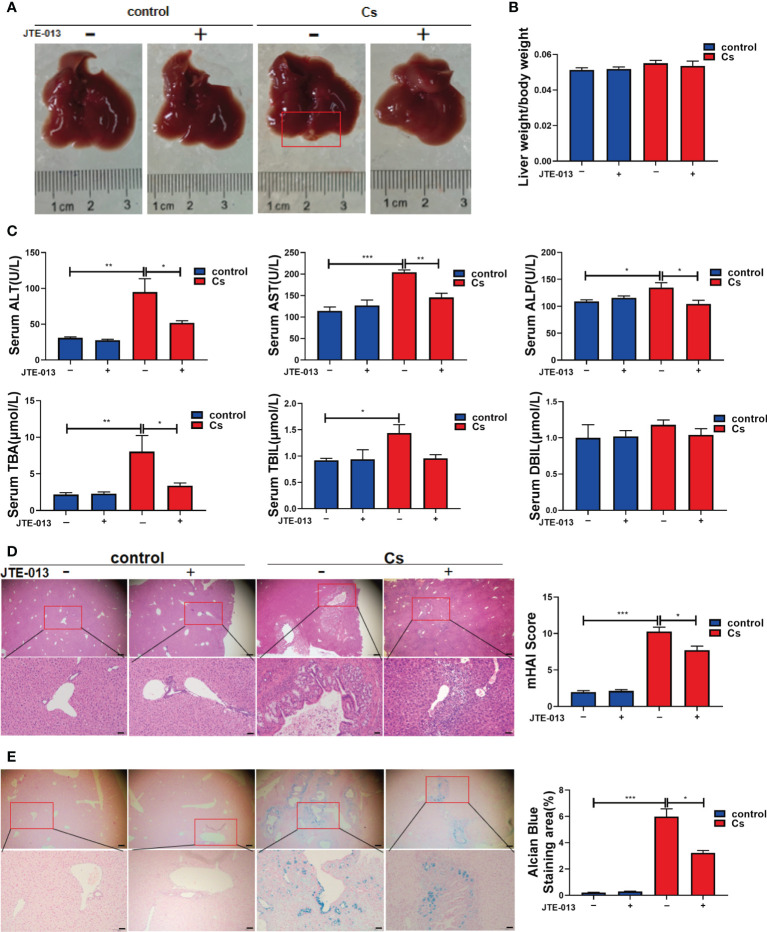
Inhibition of S1PR2 alleviated hepatobiliary damage in *C. sinensis*-infected mice. **(A)** The images of hepatobiliary injury in control and *C. sinensis*-infected mice. **(B)** The liver weight/body weight ratio in control and *C. sinensis*-infected mice. **(C)** The serum analysis of AST, ALT, ALP, TBA, DBIL, and TBIL in control and *C sinensis*-infected mice. **(D)** The H&E analysis the damage in hepatobiliary tissue of control and *C. sinensis*-infected mice under 40× and 200× microscope. **(E)** The Alcian Blue analysis the damage in hepatobiliary tissue of control and *C. sinensis*-infected mice under 40× and 200× microscope. Compared with indicated groups, *P<0.05, **P<0.01, ***P<0.001.

To explore the role of S1PR2 in hepatobiliary damage in mice caused by infection with *C. sinensis*, JTE-013 was used to block S1PR2 in *C. sinensis*-infected mice and non-infected mice. There were obvious lesions on the surface of the livers in the *C. sinensis-*infected group, compared with the non-infected group. However, blockade of S1PR2 by JTE-013 markedly reduced the lesions, compared with the *C. sinensis*-infected mice administrated with vehicle (**Figure 4A**). However, the liver weight/body weight ratio showed no significant change in the *C. sinensis*-infected group compared with the control group (**Figure 4B**). In addition, the hepatobiliary damages were more severe in *C. sinensis*-infected mice than the control group as indicated by the measurements of ALT (*P*<0.01), AST (*P*<0.001), ALP (*P*<0.05), TBA (*P*<0.01), and TBIL (*P*<0.05). However, blockade of S1PR2 by JTE-013 significantly decreased the expression level of ALT (*P*<0.05), AST (*P*<0.01), ALP (*P*<0.05), and TBA (*P*<0.05), compared with the *C. sinensis*-infected mice administrated with vehicle (**Figure 4C**). As shown by H&E staining of the hepatobiliary structure, more serious damages were observed in the *C. sinensis*-infected mice, compared with the non-infected mice, and JTE-013 treatment could alleviate the hepatobiliary damages (**Figure 4D**, *P*<0.001, *P*<0.05). Similarly, as shown by Alcian Blue staining, there were abundant blue deposits in and around the hyperplastic bile ducts in the *C. sinensis*-infected mice while the hepatobiliary damages were alleviated upon JTE-013 treatment (**Figure 4E**, *P*<0.001, *P*<0.05).

### Inhibition of S1PR2 abates periductal fibrosis caused by *C. sinensis* infection

**Figure 5:**
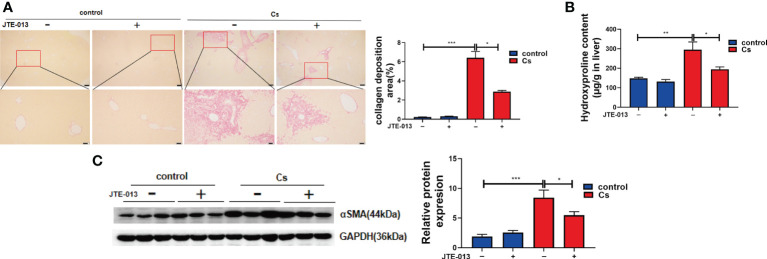
Inhibition of S1PR2 abated periductal fibrosis in BALB/C mice after *C. sinensis* infection. **(A)** The Sirius Red’s staining of the hepatobiliary tissue was carried out in the control and *C. sinensis*-infected mice under 100× and 200× microscope. **(B)** The measurement of hydroxyproline content in hepatobiliary tissue of control and *C. sinensis*-infected mice. **(C)** The protein expression of alpha-SMA in hepatobiliary tissue of control and *C. sinensis*-infected mice. Compared with indicated groups, *P<0.05, **P<0.01, ***P<0.001.

Sirius Red’s staining showed that there was very little accumulation of collagen fibers in the non-infected mice liver, and *C. sinensis* infection-induced massive ECM deposition in the liver as shown by red strips (**Figure 5A**, *P*<0.001). In contrast, after the blockade of S1PR2 by JTE-013, the accumulation of collagen fibers represented by the red strips was significantly decreased, compared with the *C. sinensis*-infected mice (**Figure 5B**, *P*<0.05). Similarly, hydroxyproline content was much higher in the *C. sinensis*-infected group than that in the non-infected group (**Figure 5B**, *P*<0.01). Although *C. sinensis* infection induced a significant increase in hydroxyproline content, the inhibition of S1PR2 significantly decreased hydroxyproline content in the liver of *C. sinensis* infected mice treated with JTE-013 (**Figure 5B**, *P* < 0.05). Besides, compared to the control group without infection, alpha-smooth muscle actin (α-SMA), increased after *C. sinensis* infection (**Figure 5C**, *P*<0.001). However, blockade of S1PR2 by JTE-013 showed a significant decrease in α-SMA expression compared to the *C. sinensis*-infected group (**Figure 5C**, *P*<0.05). These data together indicate that inhibition of S1PR2 meliorated periductal fibrosis caused by *C. sinensis* infection.

### Inhibition of S1PR2 reverses the bile duct proliferation caused by *C. sinensis* infection

**Figure 6:**
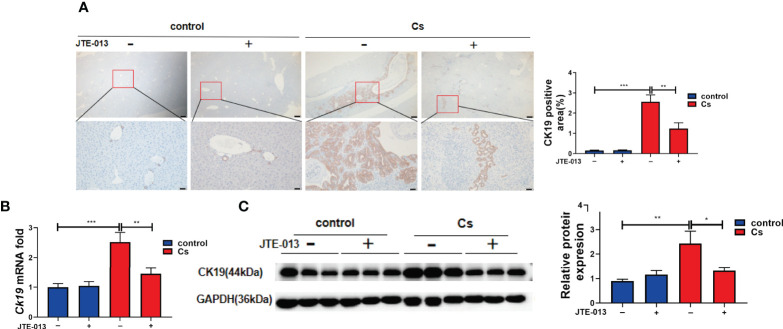
The inhibition of S1PR2 alleviated BECs proliferation in mice infected with *C. sinensis*. **(A)** The expression of BECs proliferation marker CK19 in infected mice compared to that in control mice. **(B)** The relative mRNA expression for *CK19* in hepatobiliary tissue of control and *C. sinensis*-infected mice. **(C)** The protein expression of CK19 in hepatobiliary tissue of control and *C. sinensis*-infected mice. Compared with indicated groups, *P<0.05, **P<0.01, ***P<0.001.

We found that the *C. sinensis*-infected mice showed a significant increase in the proliferation of BECs compared to the control group (**Figure 6A**, *P*<0.001). Also, compared to the mice infected with *C. sinensis*, the blockade of S1PR2 by JTE-013, reduced the proliferation of BECs obviously, as indicated by IHC staining of CK19 (**Figure 6A**, *P*<0.01). We further detected the gene expression of *Ck19* transcripts in the hepatobiliary tissue of non-infected as well as *C. sinensis*-infected by qRT-PCR. It was found that the expression of *Ck19* mRNA transcripts in the hepatobiliary tissue of *C. sinensis*-infected mice was significantly increased compared with control mice (**Figure 6B**, *P*<0.001) while the inhibition of S1PR2 in mice significantly decreased the expression of *Ck19* mRNA transcripts (**Figure 6B**, *P*<0.01). Besides, compared to the control group without infection, the protein levels of CK19 increased after *C. sinensis* infection (**Figure 6C**, *P*<0.01), however, inhibition of S1PR2 can significantly decrease the CK19 expression compared to the *C. sinensis*-infected group (**Figure 6C**, *P*<0.05). Taken together, our data suggest that inhibition of S1PR2 abates biliary injuries in BALB/c mice infected by *C. sinensis*.

### Inhibition of S1PR2 decreased the pro-inflammatory cytokines expression caused by *C. sinensis* infection

**Figure 7:**
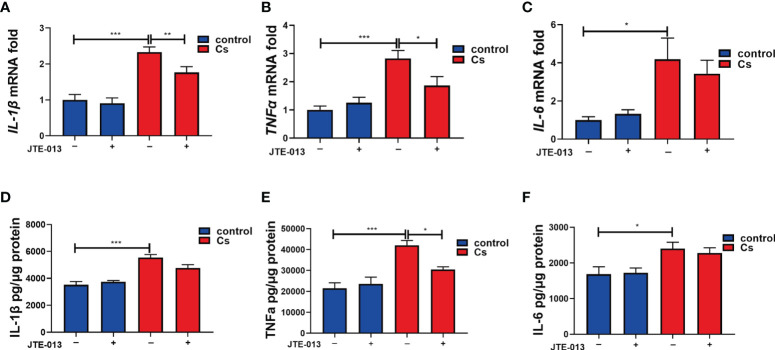
The inhibition of S1PR2 decreased the production of the pro-inflammatory cytokines after *C. sinensis* infection. **(A–C)** Measurement of the pro-inflammatory cytokines *IL-1β*, *TNFα*, and *IL-6* the relative mRNA expression in hepatobiliary tissue of control and *C. sinensis*-infected mice. **(D-F)** Measurement of the pro-inflammatory cytokines IL-1β, TNFα, and IL-6 by ELISA in hepatobiliary tissue homogenate of control and *C. sinensis*-infected mice. Compared with indicated groups, *P<0.05, **P<0.01, ***P<0.001.

We further detected the levels of the pro-inflammatory cytokines in the control and the *C. sinensis*-infected group. qRT-PCR showed that *IL-1β* (**Figure 7A**, *P*<0.001), *TNFα* (**Figure 7B**, *P*<0.001), and *IL-6* (**Figure 7C**, *P*<0.05) cytokines significantly increased after *C. sinensis* infection in the hepatobiliary tissue, while the inhibition of S1PR2 by JTE-013 significantly decreased the *IL-1β* (*P*<0.01) and *TNFα* (*P*<0.05) gene expression except for *IL-6* (**Figures 7A–C**). ELISA showed that IL-1β (*P*<0.001), TNFα (*P*<0.001), and IL-6 (*P*<0.05) cytokines significantly increased after *C. sinensis* infection in the hepatobiliary tissue, while the inhibition of S1PR2 by JTE-013 significantly decreased the TNFα (*P*<0.05) protein expression (**Figures 7D–F**). So, inhibition of S1PR2 decreased the expression of pro-inflammatory cytokines caused by *C. sinensis* infection *in vivo*.

### The blockage of S1PR2 alleviates CsESPs-induced BECs migration and pro-inflammatory cytokines production *in vitro*

**Figure 8:**
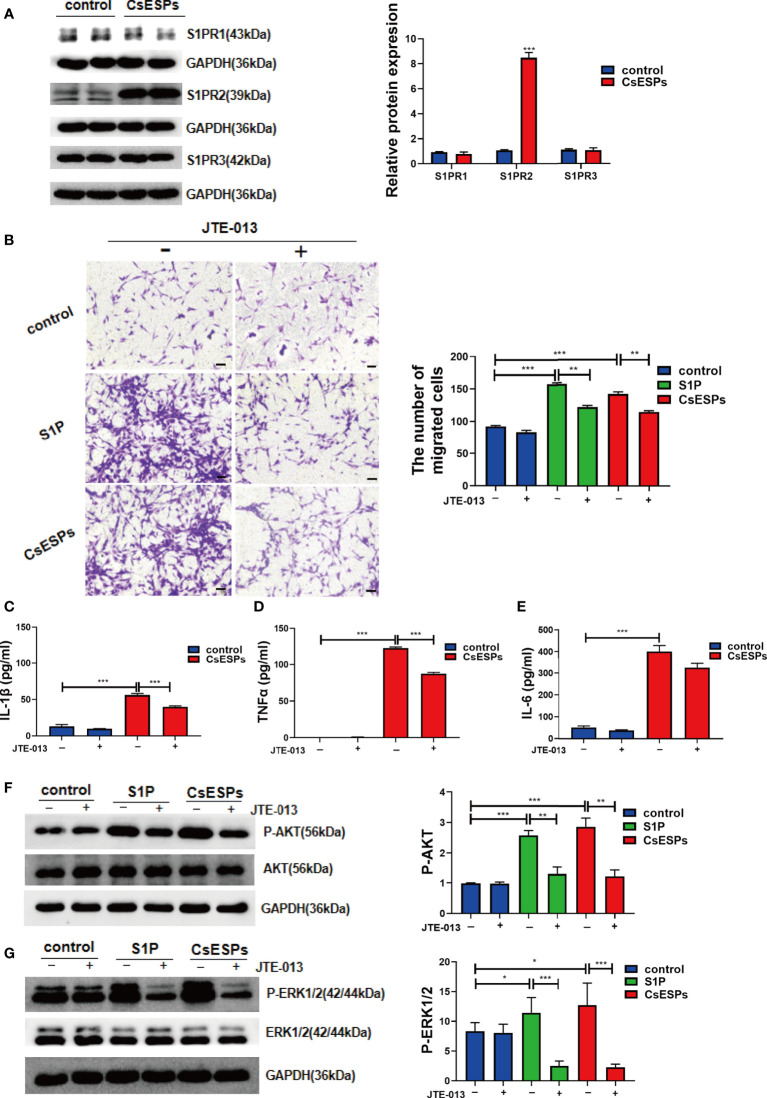
Role of S1PR2 played in CsESPs -induced migration and pro-inflammatory cytokines production of BECs. **(A)** After CsESPs stimulation, the protein levels of S1PR1, S1PR2, and S1PR3 were detected by western blotting. **(B)** Effect of JTE-013 on CsESPs/S1P-induced cell migrated in cultured BECs by transwell assay. **(C-E)** Measurement of the pro-inflammatory cytokines IL-1β, TNFα, and IL-6 the relative protein expression by ELISA in in the BECs culture supernatant. **(F, G)** BECs were pretreated with JTE-013 (10μM) for 30 minutes, then treated with vehicle control (DMSO), CsESPs (60ug/ml), or S1P (100nM). Protein levels of *P*-AKT, AKT, P-ERK1/2, and ERK1/2 were determined by western blotting. Compared with indicated groups, *P<0.05, **P<0.01, ***P<0.001.

Previous studies showed that CsESPs promotes migration and invasion (Pak et al., 2017) and increases malignant characteristics of cholangiocarcinoma cells (Won et al., 2019). To determine the effect of CsESPs on BECs and whether S1PRs are involved in this process or not, we firstly detected the expression levels of S1PRs in the BECs stimulated with CsESPs, the data showed that CsESPs stimulations significantly increased the expression of S1PR2 but not S1PR1 nor S1PR3 (**Figure 8A**, *P*<0.001). In addition, the effect of S1PR2 on CsESPs- and S1P-induced cell migration in BECs was tested by transwell assay. It showed that both CsESPs and S1P-induced cell migrations were inhibited by JTE-013 (**Figure 8B**, *P*<0.01). Additionally, we detected the levels of the pro-inflammatory cytokines of cell culture supernatant. The data showed that IL-1β (*P*<0.001), TNFα (*P*<0.001), and IL-6 (*P*<0.001) cytokines significantly increased after CsESPs treatment in the cell culture supernatant, while the inhibition of S1PR2 by JTE-013 significantly decreased the production of IL-1β (*P*<0.001) and TNFα (*P*<0.001) except for *IL-6* (**Figures 8C–E**). Meanwhile, both CsESPs and S1P significantly induced phosphorylated AKT (*P*-AKT, **Figure 8F**, *P*<0.001) and phosphorylated ERK1/2 (*P*-ERK1/2, **Figure 8G**, *P*<0.05), which were inhibited by the S1PR2 antagonist, JTE-013 (**Figures 8F, G**, *P*<0.01, *P*<0.001).

## Discussion

A major pathological consequence of *C. sinensis* infection is the injury of the hepatobiliary system. The worms and CsESPs are associated with hepatobiliary damage, inflammation, periductal fibrosis, and even the development of cholangiocarcinoma by direct and continuous contact with the final host. The liver is the central organ involved in lipid metabolism. Dysregulation of the homeostasis of certain lipid species has been implicated with hepatobiliary diseases (Nagahashi et al., 2016). Here, we characterized the major metabolic pathways and biological effects of S1PR2 during *C. sinensis* infection-induced hepatobiliary injury. Our work provided several new findings as follows: (1) we found that the production of abundant LPC and sphingolipid metabolic pathways is activated in *C. sinensis* infection; (2) S1PR2 is the dominant membrane receptor highly expressed in BECs after *C. sinensis* infection *in vitro* and *in vivo*. (3) Compared with *C. sinensis*-infected mice, we found that blockade of S1PR2 by JTE-013 alleviated hepatobiliary injuries. (4) CsESPs can activate the AKT and ERK1/2 signaling pathways, which can be inhibited by JTE-013.

Lipids, proteins and nucleic acids, are essential components of biological membranes, and the regulation of lipid metabolism is essential for the maintenance of cellular homeostasis (Bian et al., 2021). Sphingolipids, an essential lipids composition, are ubiquitous building blocks of mammal cell membranes. A large number of evidence demonstrates that sphingolipid metabolites, particularly ceramide (Castro et al., 2014) and S1P (Cartier and Hla, 2019), are signaling molecules to regulate a diverse range of cellular processes that are important in immunity, cells activation, invasion and metastasis of cancer cells (Maceyka and Spiegel, 2014; Grbcic et al., 2020; Olona et al., 2021). However, little is known of changes and regulatory direction of metabolic pathways in *C. sinensis* infection.

Lysophosphatidylcholine (LPC), a class of lipid biomolecule, has many biological functions in organisms, such as pro-inflammatory, oxidative stress, and apoptosis induction (Liu et al., 2020b). Previous *in vitro* studies have demonstrated that increased LPC is supposed to be an important etiological factor in BECs injury in pancreaticobiliary malfunction or intrahepatic cholelithiasis and inhibits BECs apoptosis, contributing to BECs senescence and carcinogenesis (Gwak et al., 2006; Fujita et al., 2014; Shimizu et al., 2015). In the present study, compared with control mice, metabolome revealed that the expression of LPC was significantly increased in *C. sinensis*-infected mice. Additionally, *Lpcat4*, *Pla2g4f*, enzymes that regulate LPC biosynthesis, increased significantly in *C. sinensis*-infected hepatobiliary tissue, indicating an altered membrane phospholipids metabolism. In addition to LPC, SHexCer, a bioactive sphingolipid, was also significantly elevated after *C. sinensis* infection. KEGG pathway analysis based on metabolomics indicates sphingolipid metabolism was involved in *C. sinensis* infection. The ceramide and sphingosine-1-phosphate (S1P) are key sphingolipid signaling molecules that regulate many pathobiological processes. In our present study, we found that multiple enzymes were involved in the regulation of ceramide and S1P. By performing metabolomics on non-infected and *C. sinensis*-infected mice, we unraveled that *C. sinensis*-infected activated the sphingolipid metabolic pathway. Our qRT-PCR data revealed that sphingolipid metabolic key genes encoding enzymes: *Cers2, Gba2*, *Gla, Asah1*, *Asah2*, *Acer2*, *Acer3*, *Neu3*, *Sgpp1*, and *Sphk1* were robustly upregulated in mRNA levels by the infection of *C. sinensis*. These data portrayed a rewired sphingolipid metabolism culminating in producing S1P in *C. sinensis-*infected hepatobiliary tissue. In these metabolic pathways, Cers2 is a major ceramide synthase responsible for *de novo* and has a critical role in liver homeostasis (Pewzner-Jung et al., 2010). Galc and Gba2 can hydrolyze glycosphingolipids (GSL) to produce ceramide (Dodge et al., 2015). Asah1, Asah2, Acer2, and Acer3 are known to catalyze ceramide into sphingosine (Coant and Hannun, 2019; Sugano et al., 2019), which could be phosphorylated by Sphk1 to form S1P (Zheng et al., 2019). Thus, these elevated enzymes may collaborate to sequentially catalyze the active metabolism from ceramide to S1P in *C. sinensis*-infected hepatobiliary tissue. In addition, compared with the control group, the protein expression of Sphk1 and S1P concentration in hepatobiliary tissue were significantly increased. Sphk1 is a key enzyme in the phosphorylation of sphingosine to S1P. Sphk1/S1P/S1PRs axis plays an important role in injuries such as fibrosis (Xiu et al., 2015), inflammation (Hou et al., 2020), tumor cell migration and invasion (Bao et al., 2012). S1P is a bioactive sphingolipid metabolite involved in many critical cellular processes through its G protein-coupled receptors S1PRs, whereas S1P can also function intracellularly as a second messenger (Cartier and Hla, 2019). So, it is speculated that the hepatobiliary injury caused by *C. sinensis* infection is correlated with the increased expression of Sphk/S1P/S1PRs.

S1PRs are a kind of G protein-coupled receptor (GPCR), which have five different subtypes: S1PR1-S1PR5. S1PRs are located in different tissues in vertebrates: S1PR1, S1PR2, and S1PR3 are ubiquitously expressed in multiple tissues; however, the expression of S1PR4 is limited to lymphatic and hematopoietic tissues, and S1PR5 is expressed in the central nervous system (Wang et al., 2018). S1PR2 is one of the five S1PRs identified so far and has been implicated in mediating different cellular functions and pathologies (Adada et al., 2013). It has been previously shown that conjugated bile acids (CBAs) activate S1PR2 in primary hepatocytes (Studer et al., 2012). Furthermore, several studies are reporting that activating S1PR2 promotes hepatic fibrosis (Yang et al., 2015), portal hypertension (Kageyama et al., 2012), BECs proliferation and liver injury in the animal models of cholestasis (Wang et al., 2017). The current study demonstrated that, compared to the control group, S1PR2 was highly expressed in *C. sinensis*-infected hepatobiliary tissue of mice and further located in bile duct tissue *in vivo*, suggesting BECs might be more sensitive to the S1PRs blockade. Similarly, compared to the S1PR1 and S1PR3, both S1PR2 mRNA levels and protein levels are highly expressed in BECs stimulated by CsESPs. However, the physiological and pathological relevance of *C. sinensis* infection-induced activation of S1PR2 in hepatobiliary injury is not clear.

In our present study, contrast with the control mice, we found that the *C. sinensis* -infected hepatobiliary tissue showed exacerbated pathological injuries, including periductal fibrosis, production of pro-inflammatory cytokines, and bile duct hyperplasia. And the data of our present study is consistent with previous reports (Na et al., 2020; Yan et al., 2020). To further investigate the role of S1PR2 in hepatobiliary injury, JTE-013, an effective and selective S1PR2 antagonist, was used. Previous studies have used JTE-013 to identify the biological effects of S1PR2 (Wang et al., 2017; Park and Im, 2019). It was reported that JTE-013 did not affect liver enzymes in serum and liver histology in normal rats (Kageyama et al., 2012). We found that S1PR2 aggravates disease pathogenesis of *C. sinensis*-induced biliary fibrosis by producing pro-inflammatory mediators, including IL-1β, TNF-α, and IL-6, in which S1PR2 antagonism with JTE-013 administration can protect bile duct cells by downregulating mRNA and protein expression of pro-inflammatory mediators. Similar pro-inflammatory roles of S1PR2 have been reported in renal ischemic injury (Park et al., 2012). In addition, our research has confirmed that the administration of the S1PR2 antagonist relieves high levels of hepatobiliary function indexes (ALP, ALT, AST, TBIL, and TBA). The H&E and Alcian Blue staining showed that the JTE-013 treatment significantly reduced the inflammatory cell infiltration around the hyperplastic bile duct and reduced the production of the mucinous substance. Furthermore, the hepatic hydroxyproline level, the deposition of extracellular matrix, and the expression of genes related to fibrogenesis significantly elevated, whereas JTE-013 treatment significantly inhibited their expressions. The proliferation of BECs is a major characteristic of *C. sinensis* infection. The expression levels of CK19, a marker of BECs, were further examined. *C. sinensis* infection significantly induced BECs proliferation, as indicated by an increase of CK-19-positive bile ducts, which could be inhibited by JTE-013. Therefore, to some extent, inhibition of S1PR2 can reduce the hepatobiliary damage caused by *C. sinensis* infection.

S1P/S1PRs signaling exerts biological functions in different organs through intracellular effector pathways (Sanchez et al., 2007). S1PR2 plays a key role in promoting NLRP3 inflammasome priming and activation *in vitro* and *in vivo*, depending on the downstream Gα (12/13)/MAPK Signaling pathway (Hou et al., 2021b). As an important downstream pathway of S1PR2, ERK1/2 signaling has been involved in many physiological and pathological processes induced *via* S1PR2. In particular, CBAs-induced activation of S1PR2-mediated cholestasis liver injury in mice and invasive growth of human CCA cells by ERK1/2 and AKT Signaling pathway (Liu et al., 2015; Wang et al., 2017). Similarly, our results showed that CsESPs-regulated S1PR2 exerted biological effects in activated BECs which seemed to be mediated by activation of AKT and ERK1/2, which was correlated with cell migration and pro-inflammatory factors’ secretion. These data suggested that *C. sinensis* infection possessed potent abilities to induce biliary inflammation by S1PR2.

In summary, *C. sinensis* infection activates S1PR2 which promotes hepatobiliary duct injury and fibrosis induced by *C. sinensis* infection. This study supports S1PR2 as a potentially new therapeutic target for treating clonorchiasis.

## Data availability statement

The original contributions presented in the study are included in the article/**Supplementary Material**. Further inquiries can be directed to the corresponding authors.

## Ethics statement

The animal study was reviewed and approved by Committee on Ethics of Animal Experiments, Xuzhou Medical University (201701w007).

## Author contributions

J-XL, Z-HJ and K-YZ conceived and designed the protocol. J-XL performed most of the experiments, analyzed the data and wrote the manuscript. ML, Y-HS, BZ, CY, G-ZY, Q-QZ, J-LW, QY, and R-XT contributed to the materials and the acquisition of data. SK and CY revised the English. All authors read and approved the final manuscript. All authors contributed to the article and approved the submitted version.

## Funding

This study was supported by grants from the National Natural Science Foundation of China (Grant Nos: 82172297 to K-YZ), the starting grants for young scientist of Xuzhou Medical University (No. D2019040), Priority Academic Program Development of Jiangsu Higher Education Institutions of China (K-YZ). The funders had no role in study design, data collection, and analysis, decision to publish, or preparation of the manuscript.

## Conflict of interest

The authors declare that the research was conducted in the absence of any commercial or financial relationships that could be construed as a potential conflict of interest.

## Publisher’s note

All claims expressed in this article are solely those of the authors and do not necessarily represent those of their affiliated organizations, or those of the publisher, the editors and the reviewers. Any product that may be evaluated in this article, or claim that may be made by its manufacturer, is not guaranteed or endorsed by the publisher.

## Glossary

**Table:**

Acer2	alkaline ceramidase 2
Acer3	alkaline ceramidase 3
AKT	protein kinase
ALP	alkaline phosphatase
ALT	alanine aminotransferase
Asah1	acid ceramidase
Asah2	neutral ceramidase
AST	aspartate aminotransferase
BECs	Biliary Epithelial Cells
CCA	cholangiocarcinoma
Cerk	ceramide kinase
Cers2	ceramide synthase 2
Cers4	ceramide synthase 4
CK-19	cytokeratin-19
Cs	Clonorchis sinensis
DBIL	Direct bilirubin
ERK1/2	extracellular signal-regulated kinase 1 and 2
Galc	galactosylceramidase
GAPDH	glyceraldehyde 3-phosphate dehydrogenase
Gba2	glucosylceramidase beta 2
Gla	galactosidases
GPCRs	G-protein-coupled receptors
H&E	hematoxylin and eosin
IHC	immunohistochemistry
LPC	lysophosphatidylcholines
Neu2	neuraminidase 2
Neu3	neuraminidase 3
RT-PCR	reverse transcriptase polymerase chain reaction
Samd8	sphingomyelin synthase-related protein 1
Sgms2	sphingomyelin synthase 2
Sgpl1	sphingosine-1-phosphate Lyase
Sgpp1	sphingosine-1-phosphate phosphatase 1
Sgpp2	sphingosine-1- phosphate phosphatase 2
Smpd2	neutral sphingomyelinase 1
Smpd3	neutral sphingomyelinase 2
Smpd4	neutral sphingomyelinase 3
Sphk1	sphingosine kinase 1
Sphk2	sphingosine kinase 2
Sptlc1	serine palmitoyltransferase 1
Sptlc3	serine palmitoyltransferase 3
S1P	sphingosine-1-phosphate
S1PR1 (EDG1)	sphingosine 1- phosphate receptor 1
S1PR2	sphingosine 1- phosphate receptor 2
S1PR3 (EDG3)	sphingosine 1- phosphate receptor 3
TBA	total bile acid
TBIL	total bilirubin
Ugcg	UDP-glucose ceramide glucosyltransferase
