# Supplementary material for: Clonorchis sinensis infection induces hepatobiliary injury via disturbing sphingolipid metabolism and activating sphingosine 1-phosphate receptor 2
Source: Front Cell Infect Microbiol. 2022 Oct 19;12:1011378. doi: 10.3389/fcimb.2022.1011378 (PMC9627039; doi:10.3389/fcimb.2022.1011378)
Supplement: Supplementary file 1 [file DataSheet_1.docx]

**Supporting information for**

***Clonorchis sinensis* infection induces** **hepatobiliary injury via promoting sphingolipid metabolism and activating sphingosine 1-phosphate receptor 2**

**Supplementary Table**

Table S1 The sequences of the primer pairs used in this study

| Target | Oligonucleotide sequence (5'–3') |  |
| --- | --- | --- |
|  | Primer sense | Primer antisense |
| *mSgpp1* | GCTCATCATCATCGGGCTTC | TCTAAGGATAGGCCCAGGGT |
| *mSgpp2* | GCCAGGCGAAGACTAGAGAT | GTGCAACATTGGCACAAAGG |
| *mNeu2* | GTCCTGCAGAAGGAGACACT | CAATCAACTCTGCGTGCTCA |
| *mNeu3* | ACTGATAGTGGTGGCTGCTT | GCAGAGGGCACTTCTGAGTA |
| *mLpcat4* | GGCAAAGGTGCTACAGAAGG | GTCAACCAAACCCTTGGCAT |
| *mPla2g4f* | ACGACAAACAGCTGAGGAGA | GAGGGCATGCCTGATAGTCT |
| *mCers2* | TCATCCCTTCTCAGTATTGGT | ATCCTTTCGCTTGACATCAG |
| *mCers4* | CCCTGAATTTGTCCCTGTA | CTTGAAGTCCTTGCGTTTG |
| *mCerk* | ATCTCCACGGGACAATAAA | GGCCATACAGGGCTTTC |
| *mAsah1* | AATAACACTTGGGTTGTCAC | TAGGATACCCAGATAACCAC |
| *mAsah2* | AGAGAGAGCAAGGTATTCTTC | ACTATTTACAAAGTGGTTGC |
| *mAcer2* | GTGTGGCATATTCTCATCTG | TAAGGGACACCAATAAAAGC |
| *mAcer3* | GATTCACTGAGGAACTTTCG | AGAGAAACTTCACTTTTGGC |
| *mGalc* | CCGATTTCCTCTTCCTTGCT | GGTTCAATATGCGACTCCAA |
| *mGba2* | CGTCCTTTGCCCTCGTC | TGCCACCACTCCACTCATC |
| *mGla* | ACCCTTTCATAAGCCCAATT | GGTCCAGCGACTTCAACAA |
| *mUgcg* | GCTTCGTGCTCTTCGTGG | TTGCCTTCTTGTTGAGGTGT |
| *mSgpl1* | GAACCGACCTCCTCAAGCT | TCATACACCCAGACTATCAGC |
| *mSptlc1* | TACGAGGCTCCAGCATACC | TCAGAACGCTCCTGCAACT |
| *mSptlc3* | ACATCCATGAGTCCCGTAG | TCCATACCTCCAATGTTCC |
| *mSmpd2* | GCCCAGTTCATCCACCAC | CCTCAGTCTCAACGAAAGC |
| *mSmpd3* | TCATGGACGTGGCCTATC | ACCTGCACCTTGAGAAACAG |
| *mSmpd4* | GGAATCTCCGATGCCTACA | ATCATTGGACCACTTGGGT |
| *mSamd8* | CAGACCTACCCACCACTCC | TAGCACAGAATCACGCCAC |
| *mSgms2* | TGGTATTGGTTGGGTTATGG | CGGGCACAGGTAACGTAGTG |
| *mSphk1* | ACAGACCATCCAAAGGTAGTTT | CTCTATTCTGTGCTCAGTCTGTC |
| *mSphk2* | GTACTCATGTTGGGCATCTT | CATACTCCACTAACTCCCCA |
| *mS1pr1* | CTCCACCGTGCTCCCGCTCTA | GGAGATGTTCTTGCGGAAGGTCAGG |
| *mS1pr2* | GGCCTAGCCAGTGCTCAGC | CCTTGGTGTAATTGTAGTGTTCCAGA |
| *mS1pr3* | ACTCTCCGGGAACATTACGAT | CCAAGACGATGAAGCTACAGG |
| *mActin* | GGCACCACACCTTCTACAATG | GTGGTGGTGAAGCTGTAGCC |
| *mTNF* | GCCTCTTCTCATTCCTGCTT | CTCCTCCACTTGGTGGTTTG |
| *mIL-1β* | AAAAGCCTCGTGCTGTCG | AGGCCACAGGTATTTTGTCG |
| *mIL-6* | GTTCTCTGGGAAATCGTGGA | GGTACTCCAGAAGACCAGAGGA |
| *mCK19* | TGACCTGGAGATGCAGATTG | CCTCAGGGCAGTAATTTCCTC |
| *mAbcb1a* | CAGTATCGACGGACAGGACA | CTTCTCGGCCATAGCGAATG |
| *hS1pr1* | TCCTCGCCATCGCCATTG | GAGAGCAGAAGCAGAGTGAAG |
| *hS1pr2* | CTGCCTTCATCACGCTCTC | GCTCTTGTCGCTGCCATAC |
| *hS1pr3* | CTACGCACGCATCTACTTCC | CACGCTCACCACAATCACC |
| *hGAPDH* | AAGGGCATCCTGGGCTAC | GTGGAGGAGTGGGTGTCG |

**Supplementary Figure and figure legends**

**Supplementary Figure 1**


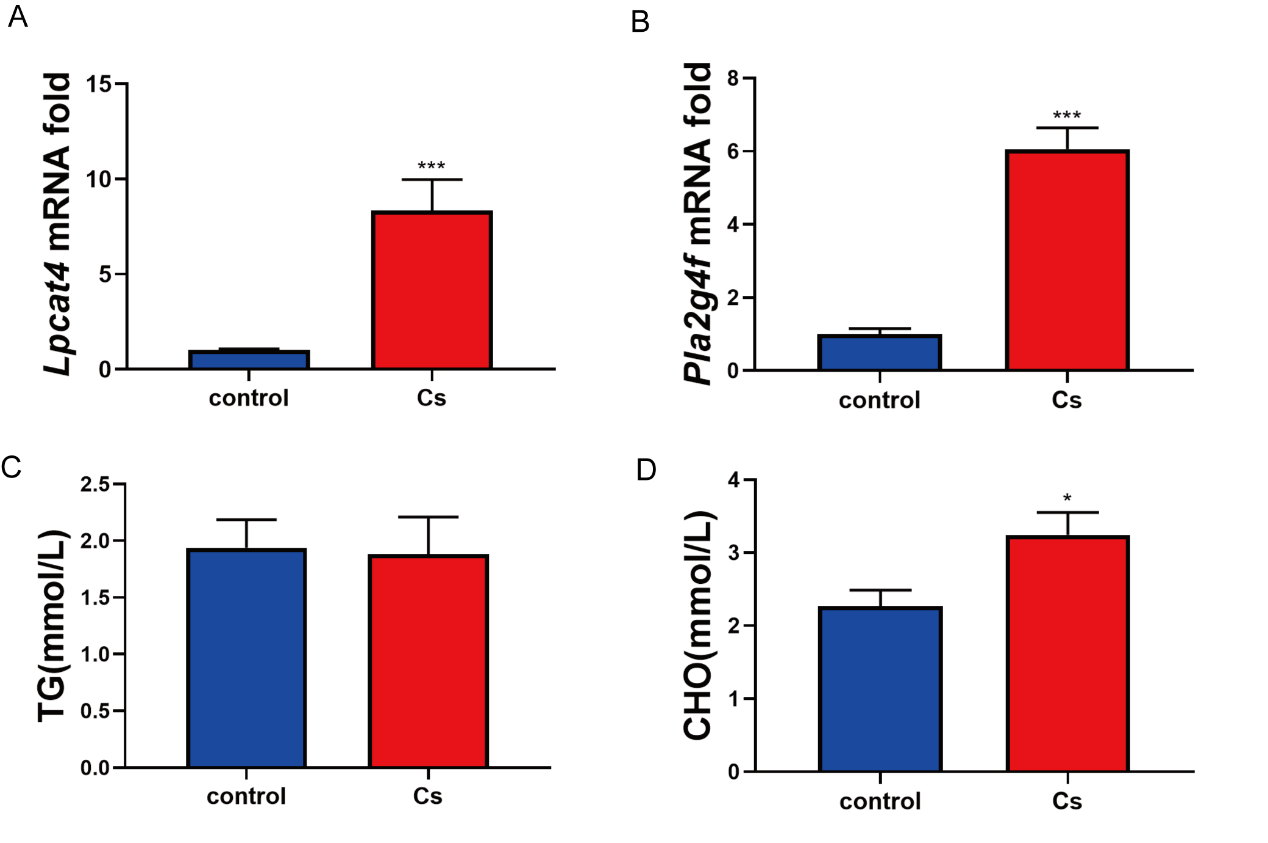


Supplementary Figure 1. **The gene expression levels of regulation LPC biosynthesis and the expression of cholesterol and triglycerides.** (A) Measurement of the *lpcat4* relative mRNA expression in liver tissue of control and *Clonorchis sinensis*-infected mice. (B) Measurement of the *pla2g4f* relative mRNA expression in liver tissue of control and *Clonorchis sinensis*-infected mice. (C) The expression of triglycerides in *Clonorchis sinensis*-infected serum. (D) The expression of cholesterol in *Clonorchis sinensis*-infected serum. Compared with indicated groups, ****P* < 0.05.

**Supplementary Materials and Methods**

**Metabolomics analysis**

Untargeted liver metabolomics analysis was performed. Liver sample preparation and metabolic profiling were performed with standard procedures in cooperation with Shanghai BIOTREE Biological Technology Co., Ltd. (Shanghai, China). Briefly, 25mg of sample was weighted to an EP tube, and 500μl extract solution (methanol: water = 3: 1, with isotopically-labelled internal standard mixture) was added. Then the samples were homogenized at 35Hz for 4min and sonicated for 5min in ice-water bath. The homogenization and sonication cycle was repeated for 3 times. Then the samples were incubated for 1h at -40℃ and centrifuged at 12000 rpm for 15min at 4℃. The resulting supernatant was transferred to a fresh glass vial for analysis. The quality control (QC) sample was prepared by mixing an equal aliquot of the supernatants from all of the samples. LC-MS/MS analyses were performed using an UHPLC system (Vanquish, Thermo Fisher Scientific) with a UPLC BEH Amide column (2.1mm×100mm, 1.7μm) coupled to Q Exactive HFX mass spectrometer (Orbitrap MS, Thermo). The mobile phase consisted of 25mmol/l ammonium acetate and 25 ammonia hydroxide in water (pH=9.75) (A) and acetonitrile (B). The auto-sampler temperature was 4℃, and the injection volume was 2μl.The QE HFX mass spectrometer was used for its ability to acquire MS/MS spectra on information-dependent acquisition (IDA) mode in the control of the acquisition software (Xcalibur, Thermo). In this mode, the acquisition software continuously evaluates the full scan MS spectrum. The ESI source conditions were set as following: sheath gas flow rate as 30 Arb, Aux gas flow rate as 25Arb, capillary temperature 350℃, full MS resolution as 60000, MS/MS resolution as 7500, collision energy as 10/30/60 in NCE mode, spray Voltage as 3.6kV (positive) or -3.2kV (negative), respectively.

The raw data were converted to the mzXML format using ProteoWizard and processed with an in-house program. Then an in-house MS2 database (BiotreeDB) was applied in metabolite annotation. In this study, peaks were detected and metabolites were left after relative standard deviation de-noising. Data was scaled and logarithmic transformed to minimize the impact of both noise and high variance of the variables. After these transformations, PCA (principle component analysis, PCA) was carried out to visualize the distribution and the grouping of the samples. In order to visualize group separation and find significantly changed metabolites, supervised orthogonal projections to latent structures-discriminate analysis (OPLS-DA) was applied. Then, a 7-fold cross validation was performed to calculate the value of R2 and Q2. To check the robustness and predictive ability of the OPLS-DA model, a 200 times permutations was further conducted. Furthermore, the value of variable importance in the projection (VIP) of the first principal component in OPLS-DA analysis was obtained. The metabolites with VIP>1 and p<0.05 (student’s t test) were considered as significantly changed metabolites. In addition, commercial databases including KEGG (http://www.genome.jp/kegg/) and MetaboAnalyst (http://www.metaboanalyst.ca/) were used for pathway enrichment analysis.
